# Transcutaneous auricular vagal nerve stimulation releases extrapineal melatonin and reduces thermal hypersensitivity in Zucker diabetic fatty rats

**DOI:** 10.3389/fnins.2022.916822

**Published:** 2022-08-11

**Authors:** Shuxing Wang, Shaoyuan Li, Xu Zhai, Peijing Rong, Jietao He, Lina Liu, Xinxin He, Wenguo Liu

**Affiliations:** ^1^School of Medicine, Foshan University, Foshan, China; ^2^Department of Physiology, Institute of Acupuncture and Moxibustion, Academy of Chinese Medical Sciences, Beijing, China

**Keywords:** extrapineal melatonin secretion, transcutaneous auricular vagal nerve stimulation, chronic pain relief, Zucker diabetic fatty rats, COVID-19 rehabilitation

## Abstract

Type 2 diabetes (T2D) is the most common comorbidity of COVID-19, and both are related to the lack of circulating melatonin. In addition, chronic pain is a common sequela of both COVID-19 and T2D. Using a neuropathic pain model produced by sciatic nerve chronic constriction injury in Zucker diabetic fatty rats, a verified preclinical genetic T2D neuropathy animal model, this study aimed to show that transcutaneous auricular vagal nerve stimulation (taVNS) could elevate plasma melatonin concentration, upregulate the expression of melatonin receptors (MTRs) in the amygdala, and relieve peripheral neuropathic pain. Furthermore, taVNS would restore melatonin levels and relieve pain even in pinealectomized rats. On the contrary, intraperitoneally injected luzindole, a melatonin receptor antagonist, would attenuate the antinociceptive effects of taVNS. In conclusion, the mechanism of the therapeutic effect of taVNS on chronic pain involves the release of extrapineal melatonin and the positive regulation of the expression of central MTRs. This beneficial efficacy should be considered during COVID-19 rehabilitation in individuals with diabetes.

## Introduction

Affecting up to 77% of patients infected with SARS-CoV-2, chronic pain is the most common sequela of COVID-19 (Kemp et al., 2020). Angiotensin-converting enzyme 2 (ACE2) is a transmembrane protein that converts angiotensin II into angiotensin 1–7 to balance the renin-angiotensin system (Rahman et al., 2021). It has been recently verified that ACE2 is the receptor and the entry port for the SARS-CoV-2 to infect host cells. Once the virus binds to ACE2, it will lead to a downregulated expression of this enzyme (Pawlikowski and Winczyk, 2021). Physiologically, ACE2 is also the escort of tryptophan transporter protein and is necessary for the successful absorption of tryptophan from the digestive tract (Pawlikowski and Winczyk, 2021). Since tryptophan is the precursor of melatonin (5-methoxy-*N*-acetyltryptamine), a neurohormone that has a short half-life of 0.56–20 min in the blood, the patient would soon exhaust it.

A growing number of studies demonstrated that melatonin modulates glucose metabolism through receptor-dependent influences on glucagon and insulin secretion (Peschke et al., 2007; Bähr et al., 2011; Nagorny et al., 2011). In pancreatic islets, the melatonin receptor type 1 (MT1) is expressed on α-cells while the type 2 (MT2) on β-cells (Peschke et al., 2007; Nagorny et al., 2011). At a physiological concentration, melatonin increases glucagon production from pancreatic α-cells (Bähr et al., 2011) but inhibits insulin production in β-cells, but with a functional phase shift following the binding of melatonin to the MT2 receptor (Stumpf et al., 2009). In the Type 2 diabetes condition, insulin secretion may lose part of this negative regulatory mechanism and result in hyperinsulinemia (Frese et al., 2009; McMullan et al., 2013). In a clinical setting, with the expectation to restore the melatonin level, physicians may prescribe melatonin tablets or injections to patients. Unfortunately, at this time, it is not known what melatonin dosage and administration time we should give to patients, mainly due to the fact that an ultra-physiological concentration of melatonin will induce a negative effect on glucose metabolism (Gerdin et al., 2004). In addition, it is necessary for melatonin to be secreted in a fluctuant manner to regulate blood glucose concentration, which is hard to reach by administrated melatonin (Ferlazzo et al., 2020).

Decreased pineal melatonin synthesis is reported in both rodents and patients with diabetes (Wajid et al., 2020). Our previous study demonstrated that transcutaneous auricular vagal nerve stimulation (taVNS) could effectively enhance central serotonergic function and reduce pain sensitivity in diabetic rats (Li et al., 2018). Since both pain sensitivity and insulin resistance are related to low plasma melatonin concentrations, it might be beneficial to elevate the circulating melatonin. It is now clear that there exist vagal efferent branches in the auricular concha. Pulses applied to the vagal branches will eventually reach the vagal afferent branches, innervating the digestive tract and stimulating the secretion of melatonin from pheochromocytes here (manuscript in preparing). Using a rodent model of diabetic neuropathic pain induced by sciatic nerve chronic constriction injury (CCI) in Zucker diabetic fatty (ZDF; *fa*/*fa*) rats, in this study, we show that taVNS would be an approach to enhance melatonin secretion in diabetic individuals with COVID-19 and the mechanism is, at least partially, due to an enhancement of extrapineal melatonin release and an upregulation of the expression of melatonin receptors (MTRs) type 1 (MT1) in the brain.

## Materials and methods

### General methodology

#### Diabetic animal model

**FIGURE 1:**
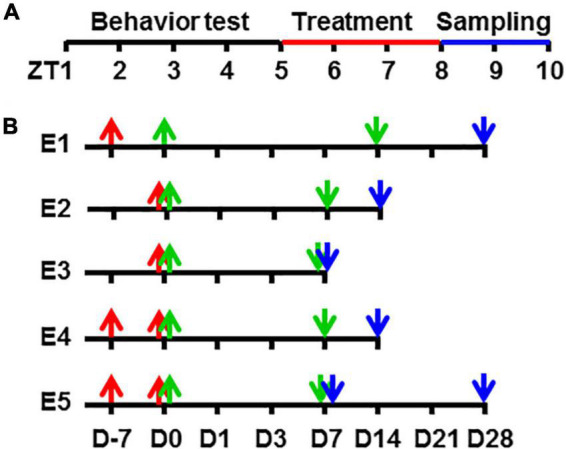
A schematic diagram of experimental procedures in zeitgeber time and time points. Showing the zeitgeber time of procedures **(A)** and the time points in each experimental group **(B)** in this study. ZT, zeitgeber time in hour [ZT0, light turn on time (7 AM)]. D, time in day; E1, Experiment 1; E2, Experiment 2, and so on.

Male ZDF (*fa*/*fa*) rats were used as the diabetic animal models and were purchased from VitalRiver Laboratories International Inc. (Beijing, China). The animal room was artificially lighted from 7 a.m. (zeitgeber time, 0, ZT0) to 7 p.m. (ZT12; Figure 1A). Littermates from the same or from a foster mother were housed in large cages until they were ready to enter the experiment procedure at 8 weeks of age. We used only mature male ZDF rats for a further detailed study to avoid a possible confounding effect from gender differences on the endogenous melatonin level and other possible hormone variations. The experimental protocol was approved by the Institutional Animal Care and Use Committee in China Academy of Chinese Medical Sciences.

#### Neuropathic pain model

The sciatic nerve CCI model was produced by loosely ligating a common sciatic nerve referring to the method of Bennett and Xie (1988). Briefly, under 2% isoflurane-inhaling anesthesia, the left side sciatic nerve was separated from surrounding tissue. Four chromic suture circles were loosely ligated around the sciatic nerve with an interval of 1 mm to each other so that only the superficial vessels of the nerve trunk were interrupted. The nerve was then put back and the wound closed with a suture clipper. The rats exhibiting postoperative neurological deficits (e.g., paralysis) or poor grooming were excluded from further experiments.

#### Electroacupuncture and time points

All the time points recorded in this study were in accordance with the taVNS occurrences, i.e., the beginning of taVNS was Day 0, and accidents that happened before this time point was recorded as the minus day. For example, D-7 meant 7 days before the beginning of taVNS, and D14 meant 14 days counted from the beginning of taVNS (Figure 1B).

For auricular concha electroacupuncture, under 2% isoflurane inhalation anesthesia, two opposite magnetic electrodes were placed over the auricular concha region of an ear, inside and outside, respectively. A procedure of 30-min transcutaneous electric nerve stimulation at a frequency of 2/15 Hz (2 and 15 Hz, switched every second) and an intensity of 2 mA was administered *via* an electrical stimulator (HANS-100, Nanjing, China). These stimulus parameters and sites have been repeatedly demonstrated to be effective in our previous study. The procedures were given in the afternoon (ZT5-8) after blood sample collection, beginning from Day 0. The acupoint selected in this study was the auricular concha of both sides, and the auricular margin was used as the sham acupoint.

#### Intraperitoneal injection

Vehicle, melatonin (60 mg/kg) or luzindole (0.01 μmol), was administered one time daily in the afternoon (ZT5-8) after blood sample collection. The dose of melatonin was based on our previous experiment and the dose of luzindole on the previous experiment. Melatonin and luzindole were purchased from Sigma Chemical Co. (St. Louis, United States) and dissolved in 5% ethanol saline (v/v) immediately before use.

#### Behavior tests

The animals were habituated to the test environment daily (a 60-min session) for 2 days before baseline testing. The testing procedure for thermal hyperalgesia was performed according to a previously published method (Hargreaves et al., 1988). The temperature was set to have the baseline latency of 12–14 s and a cutoff of 20 s. Mechanical allodynia was examined by applying a set of von Frey filaments to the plantar surface of each hindpaw, up and down depending on the withdrawal responses of the paw (Tal and Bennett, 1994). The cutoff force was 26 gm. All behavior testing was conducted between 8 a.m. and 12 p.m. (ZT1-5) before any daily drug or taVNS treatment.

#### Collection of plasma

For analyzing the concentration of melatonin in plasma, the rats were anesthetized by inhalation of 2% isoflurane in oxygen, and 0.1-ml blood samples were collected from one of the tail veins at each time point between 3 and 5 p.m. (ZT8-10) before daily treatments. The blood sample was centrifuged for 10 min at 1,000 rpm, and the plasma was collected. All plasma samples were stored at -80^°^ until use. The animals were sacrificed after the last collection of blood, and brain samples were harvested.

#### Enzyme-linked immunosorbent assay

The concentration of plasma melatonin was analyzed using an enzyme-linked immunosorbent assay (ELISA) kit (Lot# DZE30014, R&D System, Beijing, China) by Huanya Biomedicine Technology Co. Ltd. The results were read using a microplate reader (Multiskan MK3, Thermo Scientific, Beijing, China) at a wavelength of 450 nm. The plasma melatonin concentration was calculated based on the standard curve and presented in nanograms per liter (ng/L).

#### Immunohistochemical staining

The rats were anesthetized with pentobarbital (60 mg/kg, i.p.) and transcardially perfused with 200 ml of saline, followed by 400 ml of 4% paraformaldehyde in a 0.1-M phosphate buffer (PB). The brains were dissected, postfixed for 2 h, and kept in 30% sucrose in 0.1-M PB in a cold room until they sank to the bottom. Tissues were then mounted in an OCT compound and frozen on dry ice. The brain (30 μm) sections were cut on a cryostat, mounted serially onto microscope slides, and stored at -80^°^C. Immunohistochemical staining was used to detect MT1 (1:1,000, rabbit polyclonal; Abbiotec, San Diego, CA, United States). Sections were blocked with 1% goat serum in 0.3% Triton × 100 for 1 h at room temperature and incubated overnight at 4^°^C with the primary antibody. For controls, the primary antibody was omitted. The sections were then incubated for 1 h at room temperature with a corresponding Cy3-conjugated secondary antibody (1:200; JacksonImmunoResearch, West Grove, PA, United States). Brain sections were read using a LEXT OLS4000 3D Laser Measuring Microscope (Olympus), recorded using a digital camera, and processed using Adobe Photoshop.

#### Western blot

The rats were decapitated under anesthesia. Amygdala samples were collected separately and homogenized in an SDS buffer containing a mixture of proteinase inhibitors (Sigma). Protein samples were separated on SDS-PAGE gel (4–15% gradient gel; Bio-Rad, Hercules, CA, United States) and transferred to polyvinylidene difluoride filters (Millipore, Bedford, MA, United States). The filters were blocked with 3% milk and incubated overnight at 4^°^C with an MT1 primary antibody (40 kD, rabbit polyclonal, 1:500, Millipore, Billerica, MA, United States) and for 1 h at room temperature with an HRP-conjugated secondary antibody (1:10,000; Abcam, Cambridge, MA, United States). The blots were visualized in ECL solution (NEN, Boston, MA, United States) for 1 min and exposed onto hyperfilms (Amersham Biosciences) for 1–10 min. The blots were then incubated in a stripping buffer (67.5-mM Tris; pH, 6.8; 2% SDS; and 0.7% β-mercaptoethanol) for 30 min at 50^°^C and reprobed with a polyclonal rabbit anti-β-actin antibody (1:20,000; Alpha Diagnostic International, San Antonio, TX, United States) as the loading control. The Western analysis was made in triplicate. The density and the size of the bands were measured with a computer-assisted imaging analysis system and normalized against loading controls.

#### Statistical analysis

By running GraphPadInStat version 3.10 for Windows (La Jolla, CA, United States), raw data from behavior tests, ELISA, and Western blots were analyzed by using repeated measures ANOVA across testing time points to detect overall differences among treatment groups and across treatment groups to examine overall differences among testing time points. Differences were considered to be statistically significant at the level of α = 0.05.

### Experiment 1: Evaluate the therapeutic effects of transcutaneous auricular vagal nerve stimulation on chronic pain, melatonin secretion, and melatonin receptors expression

To find out whether taVNS is effective in relieving fully established neuropathic pain in ZDF rats, nociceptive behavior was evaluated in five groups of rats (*n* = 6 each): (1) naive, (2) sham CCI 1 week followed by taVNS, (3) CCI 1 week, (4) CCI 1 week followed by taVNS, and (5) CCI 1 week followed by auricular margin electroacupuncture (AMEA). Blood samples were taken at different time points for ELISA detection of the plasma melatonin level, and brain samples were collected upon sacrifice at Day 28 for Western blot detection of MT1 expression at the protein level.

### Experiment 2: Evaluate the preventive effects of transcutaneous auricular vagal nerve stimulation on chronic pain, melatonin secretion, and melatonin receptors expression

To find out whether taVNS can prevent the development of neuropathic pain after CCI and the effect of electroacupuncture on nociception behavior, melatonin secretion, and MT1 expression during the progress of neuropathic pain, five groups of ZDF rats were used (*n* = 6 each): (1) sham operation, (2) sham operation plus taVNS, (3) CCI, (4) CCI plus taVNS, and (5) CCI plus AMEA. The blood samples were collected as mentioned above for ELISA, and the brain samples taken on Day 14 for Western blot detections.

### Experiment 3: Evaluate the involvement of melatonin receptors in the function of transcutaneous auricular vagal nerve stimulation

There are mainly three types of MTRs, namely, MT1, MT2, and MT3. However, MT3 is expressed in the nucleus, while MT1 and MT2 are expressed on the cellular membrane, and MT1 is reported to be involved in the neuronal functions of the brain (Dubocovich et al., 2010). To find out whether MTRs mediate the function of taVNS and whether the blockade of MTRs modulates the release of melatonin and the expression of MTRs itself, the competitive melatonin receptor MT1/MT2 antagonist luzindole was administrated, and its effect on pain behaviors, plasma melatonin concentration, and the expression levels of MT1 were examined in four groups of ZDF rats (*n* = 6 each), including (1) a naive plus vehicle, (2) naive plus luzindole, (3) CCI, a taVNS plus vehicle, and (4) CCI, taVNS plus luzindole. Each agent or treatment was given one time daily (i.p.) in the afternoon 30–60 min before taVNS for 7 consecutive days beginning on Day 0. The blood samples were collected on Days 0 (baseline), 1, 3, and 7 after the behavior test but before any treatment. The brain samples were taken on Day 7 for Western blot detections.

### Experiment 4: Compare the effects of transcutaneous auricular vagal nerve stimulation with melatonin on pain behavior

The efficacy of taVNS was compared with that of melatonin on established and developing neuropathic pain, melatonin secretion, and MTR expression. Four groups of ZDF rats were used (*n* = 6 each): (1) CCI operation followed by taVNS 7 days later, (2) CCI operation followed by melatonin i.p. administration 7 days later, (3) CCI operation followed by taVNS immediately, and (4) CCI operation followed by melatonin i.p. administration immediately. All treatment lasted for 1 week. The rats were kept for another week to watch the lasting effects of taVNS. The brain samples were collected on Day 14 for Western blot.

### Experiment 5: Evaluate the involvement of the pineal gland in the effect of transcutaneous auricular vagal nerve stimulation

To find out the role of the pineal gland in taVNS and to explore whether there are other melatonin sources, the pain behaviors were examined in pinealectomized (Px) rats with established neuropathic pain and in rats expected to develop neuropathic pain after CCI. The pineal gland was removed from the rats according to a reported method (Maganhin et al., 2009). For the Px sham control, the skull was opened over the pineal gland but the gland was not removed. The rats exhibiting postoperative neurological deficits (e.g., paralysis) or poor grooming were excluded from further experiments. Five groups of ZDF were used (*n* = 6 each): (1) Px sham operation; (2) Px alone; (3) CCI and Px; (4) CCI and Px followed by taVNS 7 days after operation; and (5) CCI and Px followed by taVNS immediately after operation. The taVNS lasted for 7 days. Half of the rats from each group were sacrificed after the behavior test on Day 7; the brain samples were taken for Western analysis. Another half were sacrificed on Day 28 to see the long-term effects of taVNS on pain behavior and plasma melatonin in Px rats.

## Results

### The transcutaneous auricular vagal nerve stimulation was effective on both pain relief and pain progression

**FIGURE 2:**
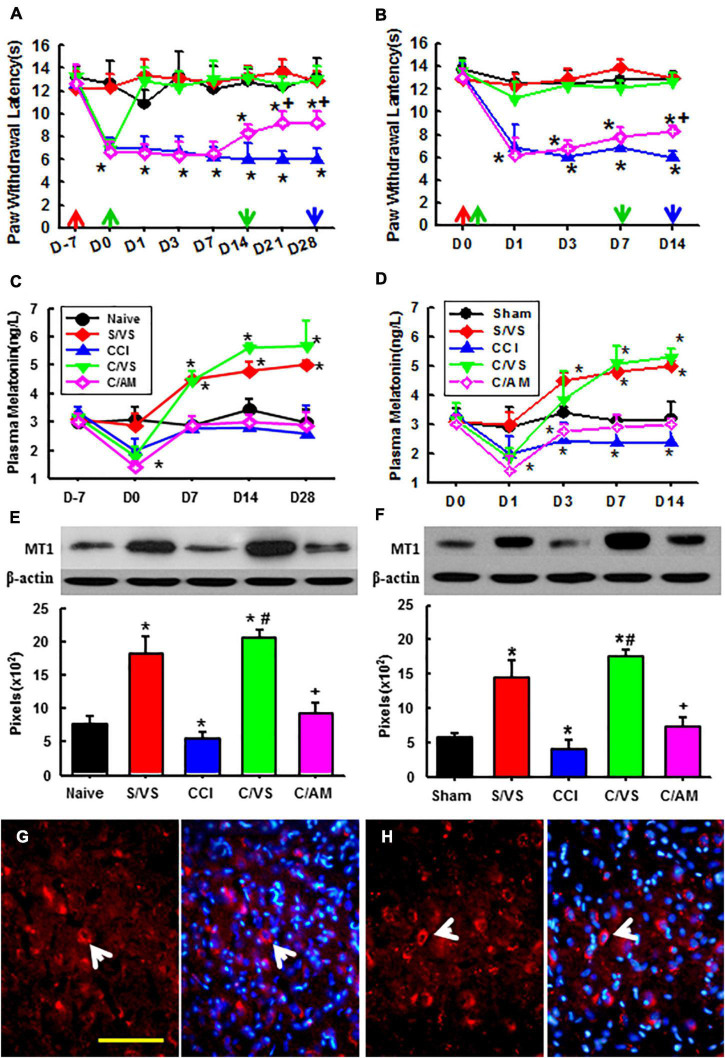
Effects of taVNS on pain behavior, melatonin level, and MT1 expression. Showing the effect of taVNS on established **(A)** and developing **(B)** thermal hyperalgesia, corresponding plasma melatonin concentration **(C,D)**, and MT1 protein expression at the last time point **(E,F)**. D, day; red arrow, CCI or sham operation; the green up arrow, treatments start; the green down arrow, treatments end; the blue arrow, sacrifice; S/VS, CCI sham operation plus taVNS; CCI, sciatic nerve chronic constriction injury; C/VS, CCI + taVNS; C/AM, CCI + AMEA. *p < 0.05, as compared with naive or sham groups on the same day and the baseline of the same group; +*p* < 0.05 as compared with S/VS, CCI, and C/VS groups; and #*p* < 0.05 as compared with CCI and C/AM groups. **(G)** The MT1 positive neurons (the arrow) in the amygdala of ZDF rats, CCI treated with AMEA, on Day 28. **(H)** The MT1 positive neurons (the arrow) in the amygdala of the ZDF rats, CCI treated with taVNS, on Day 14. Bar, 100 μm.

We watched the antinociceptive effect of taVNS on established pain first. The hyperalgesia to thermal stimulation was demonstrated 7 days after CCI operation. The taVNS treatment alleviated hyperalgesia immediately and lasted for another 2 weeks after 2 week of daily treatment (Figure 2A). The plasma melatonin concentrations and the MT1 protein expression in amygdala were both increased corresponding to the taVNS treatment (Figures 2C,E). The melatonin concentration reached a high platform at Day 14 and lasted for another 2 weeks, possibly longer, even without further taVNS session after the first 2-week daily trials. Consecutive daily taVNS sessions would not only relieve the established pain but also could prevent the development of pain after CCI if administrated starting immediately after CCI operation (Figure 2B). Similar to the efficiency in established pain, taVNS also promoted efficiency in the secretion of melatonin and in the expression of MT1 during the development of neuropathic pain (Figures 2D,F). Although, at a lower amplitude, AMEA also showed a partial analgesic effect on both established and developing pain as demonstrated by Day 14.

### Central melatonin receptor type 1 was involved in the function of transcutaneous auricular vagal nerve stimulation treatment

An immunofluorescent study found that MT1 was expressed in various brain regions. There were more MT1 positive neurons in the amygdala of the ZDF rats treated with taVNS than that in the rats treated with AMEA (Figures 2G,H).

**FIGURE 3:**
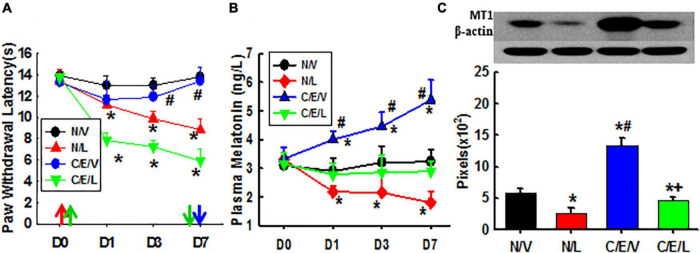
Effects of luzindole on pain behavior, the melatonin level, and MT1 expression. Showing the effect of luzindole on nociceptive thresholds to thermal stimulation and on the efficacy of taVNS **(A)**, plasma melatonin concentration **(B)**, and the expression of MT1 in amygdala (Day 7; **C)**. D, day; the red arrow, CCI operation; the green up arrow, treatments start; the green down arrow, treatments end (Day 6); the blue arrow, sacrifice; N/V, a naive plus vehicle; N/L, naive plus luzindole; C/E/V, CCI plus a taVNS plus vehicle; C/E/L, CCI plus taVNS plus luzindole. *p < 0.05, as compared with naive or the N/V group on the same day and with the baseline of the same group; #*p* < 0.05, as compared with N/L and C/E/L groups; and +*p* < 0.05, as compared with N/L.

Melatonin receptors are necessary for the therapeutic effect of taVNS as seen in Figure 3. Luzindole treatments not only reduced nociceptive thresholds to thermal stimulation in naive rats as compared with vehicle treatments but also attenuated the antinociceptive effect of taVNS. This indicates that MTRs are necessary for mediating the antinociceptive effect of taVNS. Additionally, the plasma melatonin concentration and the expression of central MT1 were also declined by luzindole treatment. This indicates that more melatonin uncombined with MTRs was eliminated and that, although luzindole competes with melatonin to combine with MTRs, it does not have the ligand-dependent positive modulation effect on the expression of MTRs as melatonin does.

**FIGURE 4:**
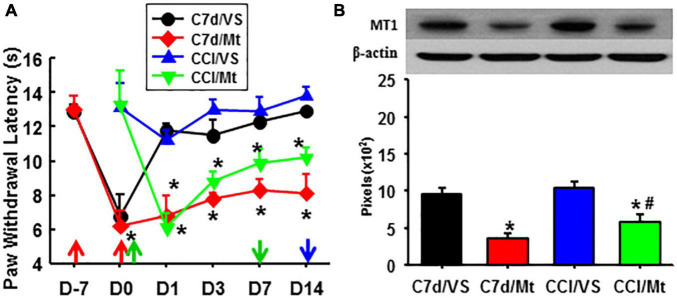
Comparison of the effects between taVNS and exogenous melatonin. **(A)** A hyperalgesia threshold to thermal stimulation. *P < 0.05 vs. CCI/VS at the same testing time points. **(B)** Expression of MT1 in the amygdala (Day 14). *P <0.05 vs. C7d/VS, #*P* <0.05 vs. CCI/VS. D, day; the red arrow, CCI operation; the green up arrow, treatments start; the green down arrow, treatments end; the blue arrow, sacrifice; C7d/VS, C7d/Mt, CCI operation followed by taVNS or melatonin i.p. injection 7 days later, respectively; CCI/VS, CCI/Mt, CCI operation followed by taVNS or melatonin i.p. injection immediately.

As compared with exogenous melatonin, taVNS effectively relieved both established and developing pain, immediately and for a long term. Exogenous melatonin, on the other side, showed a higher effect on the prevention of pain development but reduced effect on the relieving of established pain from the beginning. This might be due to a downregulated expression of MT1 during the development of pain (Figure 4).

### Transcutaneous auricular vagal nerve stimulation was effective in pain relief in pinealectomized animals

**FIGURE 5:**
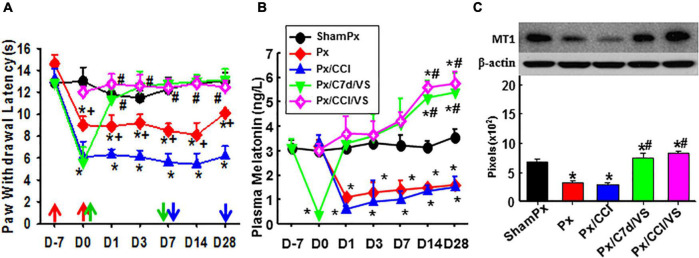
Involvement of the pineal gland in the function of taVNS. Showing the effect of taVNS on nociceptive thresholds **(A)**, plasma melatonin concentration **(B)**, and the expression of MT1 in the amygdala (Day 7; **C)** in Px rats. D, day; the red arrow, CCI, Px, or sham operation; the green up arrow, treatments start; the green down arrow, treatments end; the blue arrow, sacrifice; ShamPx, sham Px operation; Px, pinealectomized; Px/CCI, Px, and CCI; Px/C7d/VS, Px, and CCI operation followed by taVNS treatments 7 days later; Px/CCI/VS, Px, and CCI operation followed by taVNS treatments immediately. *p < 0.05, as compared with the ShamPx group on the same day and the baseline of the same group; #*p* < 0.05, as compared with Px or Px/CCI groups; and +*p* < 0.05, as compared with the Px/CCI group.

Pinealectomized rats were used to evaluate the involvement of the pineal gland in the function of taVNS (Figure 5). Px alone in the ZDF rats lowered the nociceptive threshold as compared to the sham-Px rats. taVNS reduced the pain in Px or Px plus CCI rats, beginning immediately or 1 week after CCI operation (Figure 5A). Px dramatically decreased the plasma melatonin concentration as detected at all time points in this study. However, taVNS in the Px rats restored the plasma melatonin level to the normal level immediately and even higher at 2–3 weeks after taVNS termination (Figure 5B). In addition, Px negatively modulated the expression of MT1 (Figure 5C). taVNS would positively regulate the expression of MT1 not only in the ZDF rats with an intact pineal gland (Figures 5A–C) but also in the Px ZDF rats.

## Discussion

In our body, there are parasympathetic and sympathetic autonomic nerves systems. In a night-sleeping state, the balance of the counterparts will be shifted toward an enhanced parasympathetic activity, concomitant with a reduction of the sympathetic tone (DeBenedittis et al., 1994). This autonomic imbalance can also be induced by vagus nerve stimulation (VNS), which stimulates the afferent vagus fibers, especially in the auricular concha area (La Marca et al., 2010). Although no report showed that stimulation to the parasympathetic system can enhance melatonin secretion, some pieces of evidence do demonstrate that light increases the sympathetic activity and simultaneously suppresses the vagal parasympathetic activity; both can be dose dependently attenuated by melatonin (Mutoh et al., 2003). Since bright light during the day represses melatonin secretion and due to the fact that melatonin is naturally secreted during the night, it is very likely that VNS can increase melatonin secretion. Additionally, increased sympathetic and decreased parasympathetic tones have been shown in the leptin receptor deficient (*db/db*) mice (Goncalves et al., 2009), which are genetically equivalent to the leptin receptor-deficient ZDF (*fa/fa*) rats used in this study. Combining the literature with our study results that the taVNS functionally increases the plasma melatonin concentration in ZDF rats, we conclude that taVNS stimulates the parasympathetic system and promotes the release of melatonin.

Melatonin is believed to be secreted only by the pineal gland. Interestingly, taVNS even stimulated the melatonin secretion in the Px rats. It implies that taVNS functions through the modulation of melatonin secretion from sources other than the pineal gland. According to recent reports, melatonin can be secreted also from the retina, the digestive tract, the bone marrow, the skin, kidneys, the ovaries, the testis, circulating leukocytes, and many other sources in the vertebrate species (Esposito and Cuzzocrea, 2010). It is noteworthy that these extrapineal melatonin sources have a large total volume, and some of them have high levels of melatonin, such as the melatonin pools of the gastrointestinal tract (Bubenik, 2002) and the bone marrow (Conti et al., 2000). The concentration of melatonin in both pools surpasses blood melatonin levels by 2 to 3 orders of magnitude, and the gastrointestinal tract alone secretes over 400 as much melatonin as the pineal gland (Werbach, 2008). In the gastrointestinal tract, melatonin secretes from the enterochromaffin cells, which contain precursors of melatonin (5-oxytryptophane, tryptamine, serotonin, and mexamine) and an increase in numbers after pinealectomy (Kim et al., 2012). For the release of melatonin from these pools upon and after taVNS, another beneficial key factor is that these extrapineal sources are innervated by the vegetative nervous system, and their release of melatonin is independent of photoperiodic direct regulation (Conti et al., 2000; Bubenik, 2002; Werbach, 2008).

If the therapeutic effect of taVNS on chronic pain is functionally mediated through the enhanced secretion of extrapineal melatonin as shown in the current study, one may expect that other melatonin enhancement methodologies also have therapeutic effects on pain or other illnesses lacking melatonin and that complimentarily administered melatonin has the same therapeutic effects as taVNS. The hypothesis may be partially true. On the one hand, melatonin and acupuncture are both effective for the treatment of circadian phase disorders that affect sleep (Gooneratne, 2008). Acupuncture and acupressure are also beneficial in ameliorating insomnia (Nordio and Romanelli, 2008) and in improving circadian rhythms of blood pressure in patients (Kim et al., 2012). On the other hand, exogenous melatonin may not relieve chronic pain as effectively as taVNS does for the reasons of affinity and metabolism property of both MTRs and melatonin – the MTRs are high-affinity binding sites, but the expression of these G-protein-coupled 7 transmembrane subunits may be ligands dependent; any free melatonin that is unbounded to MTRs will be rapidly distributed (serum half-life, 0.5–5.6 min) and eliminated (Iguchi et al., 1982; Paul et al., 1999). In this study, the taVNS session was 30 min daily. If we suppose that only melatonin secretion is involved, the efficacy of taVNS may also be short term. The long-term beneficial efficiency of taVNS may be attributed to a lasting release of high-level melatonin from extrapineal pools and the upregulated expression of MTRs. However, if the MTRs are combined with luzindole, taVNS-promoted extra melatonin may be eliminated quickly and may not work properly to the hyperalgesia and the expression of MTRs, as shown in our results.

Taken together, a cascade of events may occur upon taVNS stimulation: (i) taVNS stimulates the vagal afferent terminals located in the auricular concha, thus exciting the parasympathetic system; (ii) the parasympathetic signal conducts, directly or relayed through the central nervous system, to the efferent fibers innervating the gastrointestinal tract or other extrapineal melatonin sources, thus promoting the synthesis and release of extrapineal melatonin; (iii) the increased secretion of melatonin, in turn, excites the parasympathetic system, thus forming a beneficial cycle; and (iv) accumulated effect of taVNS one time daily for consecutive 7 days or over reliefs of chronic pain. Although we verified that taVNS is effective in diabetic and neuropathic chronic pain animal models (Li et al., 2018), it may be effective for all chronic pain since melatonin is a strong antioxidant in general and has an analgesic effect on chronic pain by itself (Wang et al., 2012). All these beneficial effects might be helpful to individuals with diabetes as well as individuals with COVID-19 lacking circulating melatonin. We believe that all of the results to date about the VNS and its enhancing effect on melatonin release are obtained in preclinical studies in which animals were anesthetized. Although anesthesia is helpful in precluding unwanted factors such as anxiety and distress, it raises the question of whether melatonin releases can be enhanced in a conscious state.

In this study, electrostimulation at the auricular margin elicited partial efficacy of taVNS. The reason may be that some of the stimulated currents and/or electromagnetic fields acted at the vagal terminals nearby in the auricular concha.

The ZDF rats are genetically deficient in leptin receptor expression. Both the leptin and the leptin receptor are involved in nociceptive modulation through the activation of *N*-methyl-*D*-aspartic acid (NMDA)-induced currents and through the positive modulation of NMDA receptor subunit 1 (NR1) expression (Tian et al., 2011). The plasma leptin concentration of normal humans is about 8.0 ng/ml, whereas, in the case of leptin receptor deficiency, which is the equivalent to the leptin receptor-deficient *db/db* mice, the value may reach 500–700 ng/ml (Sone and Osamura, 2001). This may explain why the ZDF rats are more sensitive to nociceptive stimulation. However, in normal rats and in high fat-fed induced obesity rats, melatonin counteracted the secretion and function of leptin (Ríos-Lugo et al., 2010). In this study, the taVNS-enhanced central melatoninergic function by promoting extrapineal melatonin release and upregulating central MT1 expression, thus might, in turn, relieve chronic pain by counteracting the function of leptin, inhibiting the NMDA-induced current, and downregulating the expression of NMDA receptor NR1 (Tian et al., 2011).

## Data availability statement

The original contributions presented in the study are included in the article, further inquiries can be directed to the corresponding author.

## Ethics statement

The animal study was reviewed and approved by Chinese Academy of Chinese Medicine.

## Author contributions

XZ, SL, JH, LL, XH, and WL carried out the experiments and collected the data. SW and PR contributed to the conception of idea, design of experiments, and analysis and interpretation of data, wrote the manuscript, and revised and approved the final version of the manuscript. All authors contributed to the article and approved the submitted version.
